# Effects of Eicosapentaenoic Acid and Docosahexaenoic Acid on Uncoupling Protein 3 Gene Expression in C_2_C_12_ Muscle Cells

**DOI:** 10.3390/nu5051660

**Published:** 2013-05-21

**Authors:** Mak-Soon Lee, In-Hwan Kim, Yangha Kim

**Affiliations:** 1 Department of Nutritional Science and Food Management, Ewha Womans University, Seoul 120-750, Korea; E-Mail: troph@hanmail.net; 2 Department of Food and Nutrition, College of Health Sciences, Korea University, Seoul 136-703, Korea; E-Mail: k610in@korea.ac.kr

**Keywords:** eicosapentaenoic acid, docosahexaenoic acid, uncoupling protein 3, AMP-activated protein kinase

## Abstract

Uncoupling protein 3 (UCP3) is a mitochondrial membrane transporter that is expressed mainly in skeletal muscle where it plays an important role in energy expenditure and fat oxidation. In this study, we investigated the effects of eicosapentaenoic acid (EPA) and docosahexaenoic acid (DHA) on UCP3 gene expression in C_2_C_12_ muscle cells. EPA and DHA up-regulated UCP3 mRNA level in a dose-dependent manner and similarly increased UCP3 promoter activity in C_2_C_12_ muscle cells. To determine whether AMP-activated protein kinase (AMPK) signaling may also directly regulate UCP3 expression, 5′-amino-4-imidazolecarboxamide-ribonucleoside (AICAR), an AMP analog that activates AMPK, was treated in C_2_C_12_ muscle cells. AICAR showed additive effects with EPA or DHA on the UCP3 promoter activation. These results indicate that EPA and DHA directly regulate the gene expression of UCP3, potentially through AMPK-mediated pathway in C2C12 muscle cells.

## 1. Introduction

Uncoupling protein (UCP) 1, 2, and 3 are mitochondrial inner membrane proteins that allow dissipation of part of the proton electrochemical gradient generated by the electron transfer chain across the mitochondrial inner membrane, increasing heat production by uncoupling respiration from adenosine triphosphate (ATP) synthesis [1]. The amino acid sequence of UCP1 is 59% and 57% identical to the sequence of UCP2 and UCP3, respectively [2]. UCP1 is expressed mainly in brown adipose tissue, which plays an important role in the maintenance of energy balance and thermogenesis induced by cold [3]. UCP2 is widely expressed in several tissues of the body, whereas UCP3 is expressed at high levels in skeletal muscle as well as in brown adipose tissue (BAT) [4]. UCP2 and UCP3 are not generally responsible for thermogenesis, but nonetheless they might be significantly thermogenic when activated fully by endogenous or exogenous environmental effectors [5]. Skeletal muscle plays important role in the energy expenditure by activation of uncoupling proteins.

A recent study reported that mice lacking UCP3 show greater fat gain induced by a long-term high-fat diet compared to wild-type mice, implicating that UCP3 is involved in the protection from fat gain induced by high-fat feeding [6]. UCP3 gene expression of skeletal muscle tissue *in vivo* was induced by dietary fish oil and docosahexaenoic acid (DHA) [7,8]. UCP3 gene expression was also up-regulated by oleic acid and a specific peroxisome proliferator-activated receptor (PPAR) gamma ligand, BRL49653 in C_2_C_12_ myotubes [9]. However, the direct effects of eicosapentaenoic acid (EPA) and DHA on the UCP3 gene expression *in vitro* remains largely unresolved. 

AMPK is a regulator of cell metabolism that is activated by a low cellular energy charge, which leads to a rise in the cellular AMP:ATP ratio [10]. AMPK activation is suggested as a critical component of both inhibition of lipogenesis and increase of thermogenesis [11]. EPA has been reported to activate AMPK in 3T3 adipocytes [12]. Activation of AMPK is associated with induction of UCP3 gene expression in rat skeletal muscle [13].

In the present study, we examined the effects of EPA and DHA in the regulation of UCP3 expression in C_2_C_12_ muscle cells. Furthermore, we investigated whether EPA or DHA on AICAR-induced AMPK activation synergistically enhance UCP3 expression in C_2_C_12_ muscle cells. 

## 2. Experimental Section

### 2.1. Reagents

EPA (C20:5, *n*-3), DHA (C22:6_,_ *n*-3), butylated hydroxytoluene (BHT), α-tocopherol, bovine serum albumin (BSA), and 5-aminoimidazol-4-carboxamide 1 β-d-ribonucleoside (AICAR) were purchased from Sigma-Aldrich (St. Louis, MO, USA). C_2_C_12_ mouse muscle cell line was obtained from American Type Culture Collection (Manassas, VA, USA). Dulbecco’s modified Eagle’s medium (DMEM), glutamine, penicillin-streptomycin, fetal bovine serum (FBS), horse serum, lipofectamine 2000, and TRIzol reagent were obtained from Invitrogen (Carlsbad, CA, USA). A cell count kit-8 (CCK-8) was purchased from Dojindo Laboratories (Kumamoto, Japan). M-MLV reverse transcriptase, pGEM^®^ T easy vector, pGL3 basic vector, and luciferase reporter assay system kit were purchased from Promega (Madison, WI, USA). pCMV-β galactosidase and pEGFP-N3 vector were obtained from Clontech (Palo Alto, CA, USA). Universal SYBR Green PCR Master Mix was purchased from Qiagen (Chatsworth, CA, USA). 

### 2.2. Cell Culture

Mouse C_2_C_12_ myoblasts were cultured in DMEM Medium supplemented with 10% FBS, 100 units/mL penicillin-streptomycin at 37 °C in a 5% CO_2_ atmosphere. When C_2_C_12_ cells reached to 90% confluence, differentiation was induced by incubation for 5 days with DMEM medium containing 2% horse serum. For RNA isolation, the differentiated C_2_C_12_ muscle cells were treated with 0 (control), 1, 10, or 50 μM of EPA or DHA in serum-free medium containing 1% BSA for 24 h, respectively. During tests of the treatments (*n* = 3), all measurements were performed in triplicate. 

### 2.3. Preparation of EPA and DHA

EPA (C20:5, *n*-3) and DHA (C22:6, *n*-3) were dissolved in 95% ethanol, and combined with 7.5% bovine serum albumin by stirring for 1 h at 37 °C. Fatty acid/BSA complex was made by conjugating the fatty acid to BSA to make a working concentration of 3 mM fatty acid prior to use. BSA solution containing 0.1% BHT and 20 μM α-tocopherol without fatty acid was used as control. 

### 2.4. Cytotoxicity Assay

Cell viability was determined by the WST-8 [2-(2-methoxy-4-nitropheyl)-3-(4-nitrophenyl)-5-(2,4-dinitrophenyl)-2*H*-tetrazolium, monosodium salt] assay, using a CCK-8 kit according to the manufacturer’s instructions. The assay is based on the cleavage of the WST-8 tetrazolium salt to formazan by cellular mitochondrial dehydrogenase. Cell viability was determined following culture in 96-well plates at a seeding density of 10^4^ cells/well. Cells were treated with 0 (control), 1, 5, 10, 20, 50, 100, 200, or 500 µM of EPA or DHA for 6, 24, or 48 h at 37 °C. WST-8/1-methoxy-phenazine methosulfate solution was added to each well and incubated for 3 h at 37 °C. The absorbance at 450 nm was measured using a Varioskan plate reader (Thermo Electron, Waltham, MA, USA). Values are expressed as a percentage of control cells without EPA or DHA.

### 2.5. Real-Time qRT-PCR

Total RNA was extracted from cells using TRIzol Reagent. The corresponding cDNA was synthesized from 4 μg of RNA using M-MLV reverse transcriptase. After cDNA synthesis, real-time qRT-PCR was performed using Universal SYBR Green PCR Master Mix on a fluorometric thermal cycler (Rotor-Gene 2000, Corbett Research, Mortlake, Australia). Primers were designed using an online program [14]. The sequences of the sense and antisense primers used for amplification were as follows: UCP3, 5′-TGTTTACTGACAACTTCCCC-3′ and 5′-TCATGTATCGGGTCTTTACC-3′; β-actin, 5′-GTTGCCAATAGTGATGACCT-3′ and 5′-GGACCTGACAGACTACCTCA-3′. The ^∆∆^Ct method was used for relative quantification. The ^∆∆^Ct value for each sample was determined by calculating the difference between the Ct value of the target gene and the Ct value of the β-actin reference gene. The normalized level of expression of the target gene in each sample was calculated using the formula 2^−∆∆Ct^. Values were expressed as fold of the control.

### 2.6. Construction of Human Uncoupling Protein 3 (UCP3)/Luc Reporter Gene

The human UCP3 gene promoter from −1790 bp to +52 bp was generated by PCR using human genomic DNA. The 5′-primer, bearing a *Mlu* I site, was 5′-ACGCGTATGGGCTGGCCTCAGGACATG-3′ and the 3′-primer, bearing a *Xho* I site, was 5′-CTCGAGTAGGGCTCCATCCCAGGAGGT-3′. Amplification of the UCP3 promoter consisted of 95 °C for 15 min followed by 35 cycles of 95 °C for 1 min, 64 °C for 2 min, and 70 °C for 1 min. The UCP3 promoter fragment (−1790/+52) was subcloned into a pGEM-T easy vector. The sequence information was deposited with GenBank (Accession no. AF208501). The UCP3 promoter fragment, corresponding to −1790 to +52 bp, was inserted into the pGL3 basic vector that includes luciferase as a reporter gene.

### 2.7. Transfection and Luciferase Activity

Transfection experiments were carried out with the Lipofectamin 2000 according to the manufacturer’s instructions. The plasmids used were 2 μg of UCP3/Luc reporter gene and 1 μg of pCMV-β galactosidase as an internal standard for the adjustment of transfection efficiency. The pGL3-basic vector was used as a vector control. Cells were incubated for 40 h after transfection and treated with 0 (control), 1, 10, or 50 μM of EPA or DHA and 0.25 mM of AICAR in serum-free medium containing 1% BSA. Transfection efficiency was calculated by measuring GFP-positive cells. Transfected cells using pEGFP-N3 vector were examined by fluorescence microscopy (Olympus Corp., Tokyo, Japan), at a magnification of 40×, after transfection. 

For luciferase assay, cells were washed with PBS and harvested with Luciferase Cell Culture Lysis Reagent. UCP3 promoter activity in cells was measured with the Luciferase Reporter Assay System using a TD 20/20 luminometer (Turner Designs, Sunnyvale, CA, USA). β-galactosidase activity was assayed enzymatically using *o-*nitrophenyl-β-d-galactopyranoside as a substrate. Luciferase activity was calculated in relative light units and normalized to β-galactosidase activity. 

### 2.8. Statistical Analysis

Values are expressed as means ± standard errors (SE). Statistical analyses were performed using SPSS software version 19 (Chicago, IL, USA). Significant differences among treatment groups were analyzed using a one-way analysis of variance followed by *post hoc* Tukey’s multiple comparison tests. *P* < 0.05 was taken to indicate a significant difference.

## 3. Results

### 3.1. Effects of EPA and DHA on Cell Viability of Muscle Cells

**Figure 1:**
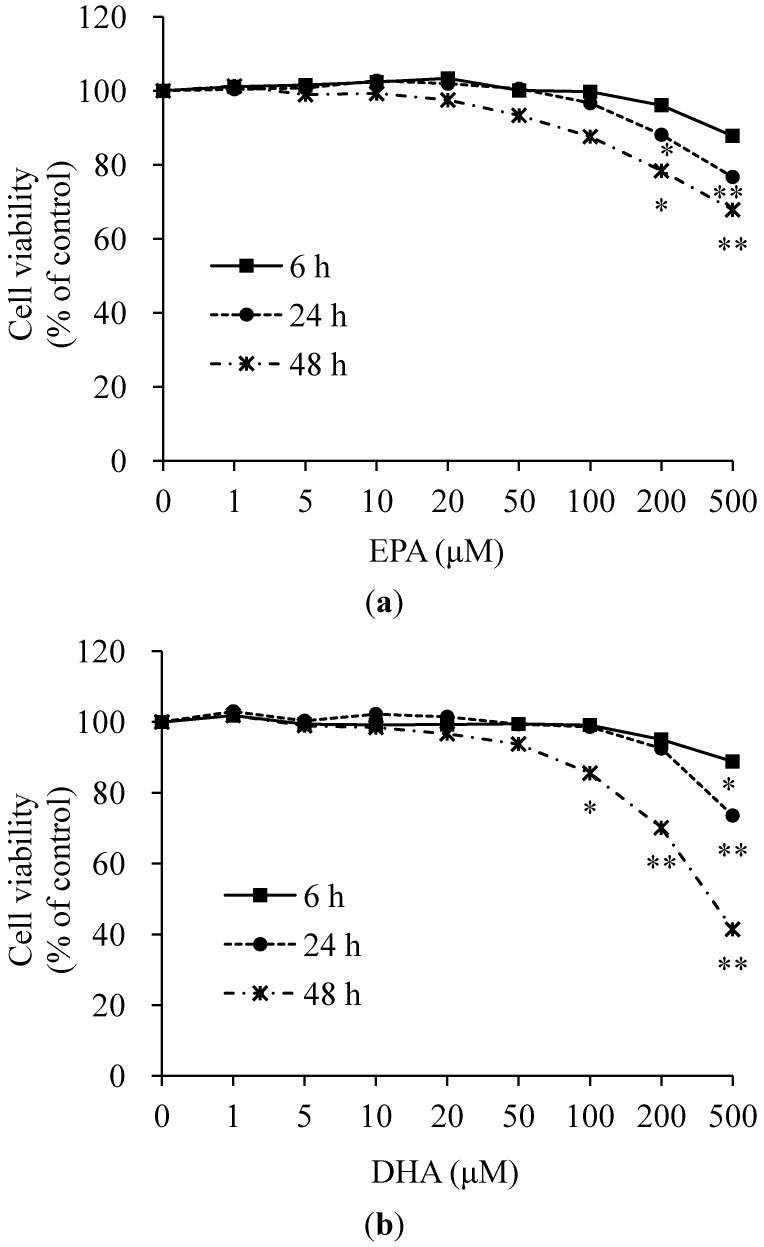
Effects of eicosapentaenoic acid (EPA) (**a**) and docosahexaenoic acid (DHA) (**b**) on the cell viability of muscle cells. Cells were treated with 0 (control), 1, 5, 10, 20, 50, 100, 200, or 500 µM of EPA or DHA, and incubated for 6, 24, or 48 h. Cell viability was determined using the WST-8 assay. Values are expressed as mean ± SE (*n* = 3) of three independent experiments. * *P* < 0.05 and ** *P* < 0.01 *versus* control treatment.

We investigated the potential cytotoxic effects of EPA and DHA on C_2_C_12_ muscle cells. Cells were differentiated for five days and then cultured in various concentrations of EPA and DHA for 6, 24, and 48 h at 37 °C. Toxicity was unaffected at 1 or 50 µM of EPA or DHA after 6, 24, and 48 h incubation. In contrast, high doses (100–500 µM) of EPA or DHA decreased viability by 3.3%–23.3% or 1.4%–26.4% at 24 h, and by 12.4%–32.1% or 14.5%–58.6% at 48 h incubation, respectively, compared to an untreated control (Figure 1). Thus, EPA and DHA were not toxic to the cell at concentrations below 50 µM and period of treatment evaluated. 

### 3.2. Effects of EPA and DHA on UCP3 Expression

**Figure 2:**
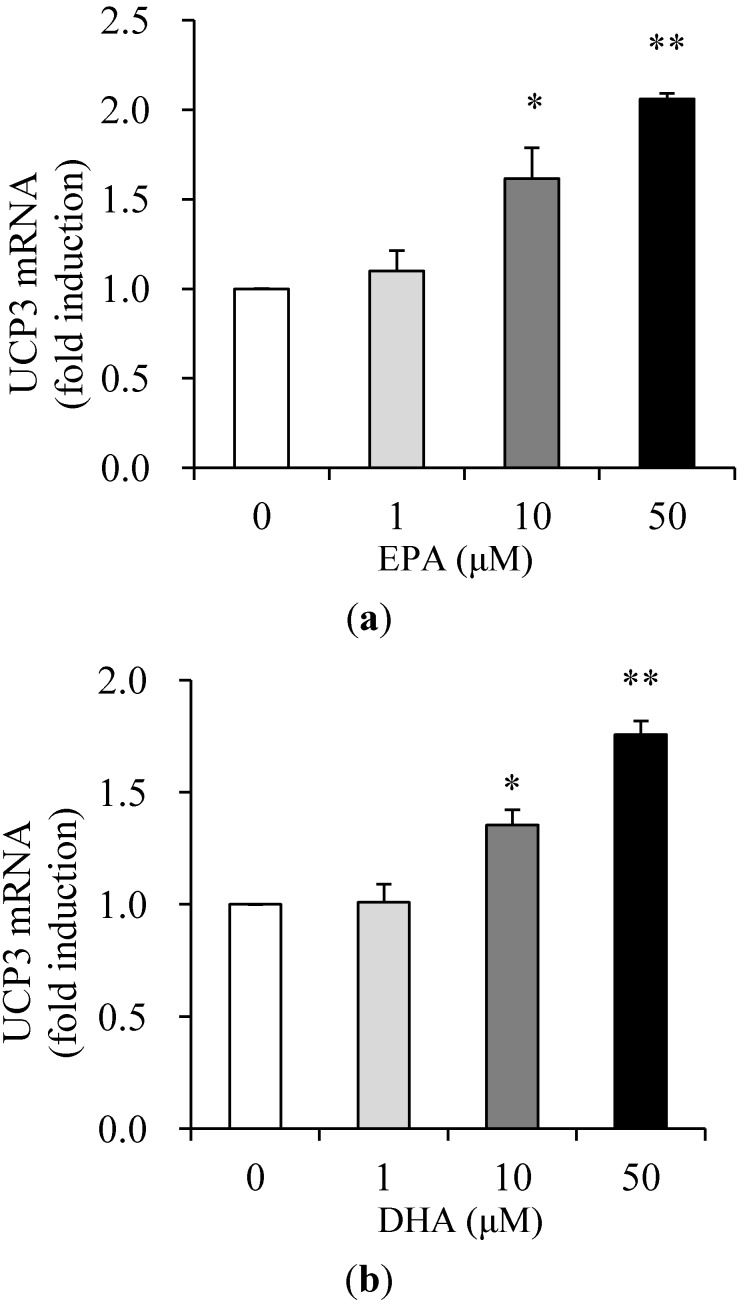
Effects of EPA and DHA on the mRNA level of uncoupling protein 3 (UCP3) in muscle cells. Differentiated C_2_C_12_ cells were treated in 1% BSA serum-free medium with concentrations of EPA (**a**) and DHA (**b**) ranging from 0 (control) to 50 μM for 24 h. The amounts of UCP3 mRNA were measured by quantitative real-time RT-PCR and are expressed as fold-change over control. Values are expressed as mean ± SE (*n* = 3) of three independent experiments. * *P* < 0.05 and **P < 0.01 *versus* control treatment.

In order to assess whether EPA and DHA directly regulate UCP3 gene expression, differentiated C_2_C_12_ cells were treated in 1% BSA serum-free medium with different concentrations (0, 1, 10, or 50 μM) of EPA or DHA for 24 h. The mRNA levels of UCP3 were examined by quantitative real-time RT-PCR. UCP3 mRNA levels were significantly up-regulated, 1.6- and 2.0-fold, in the presence of 10 and 50 μM of EPA, respectively, compared to untreated control (Figure 2a), and by 1.4- and 1.8-fold in the presence of 10 and 50 μM of DHA (Figure 2b).

### 3.3. Effects of EPA and DHA on UCP3 Promoter Activity

**Figure 3:**
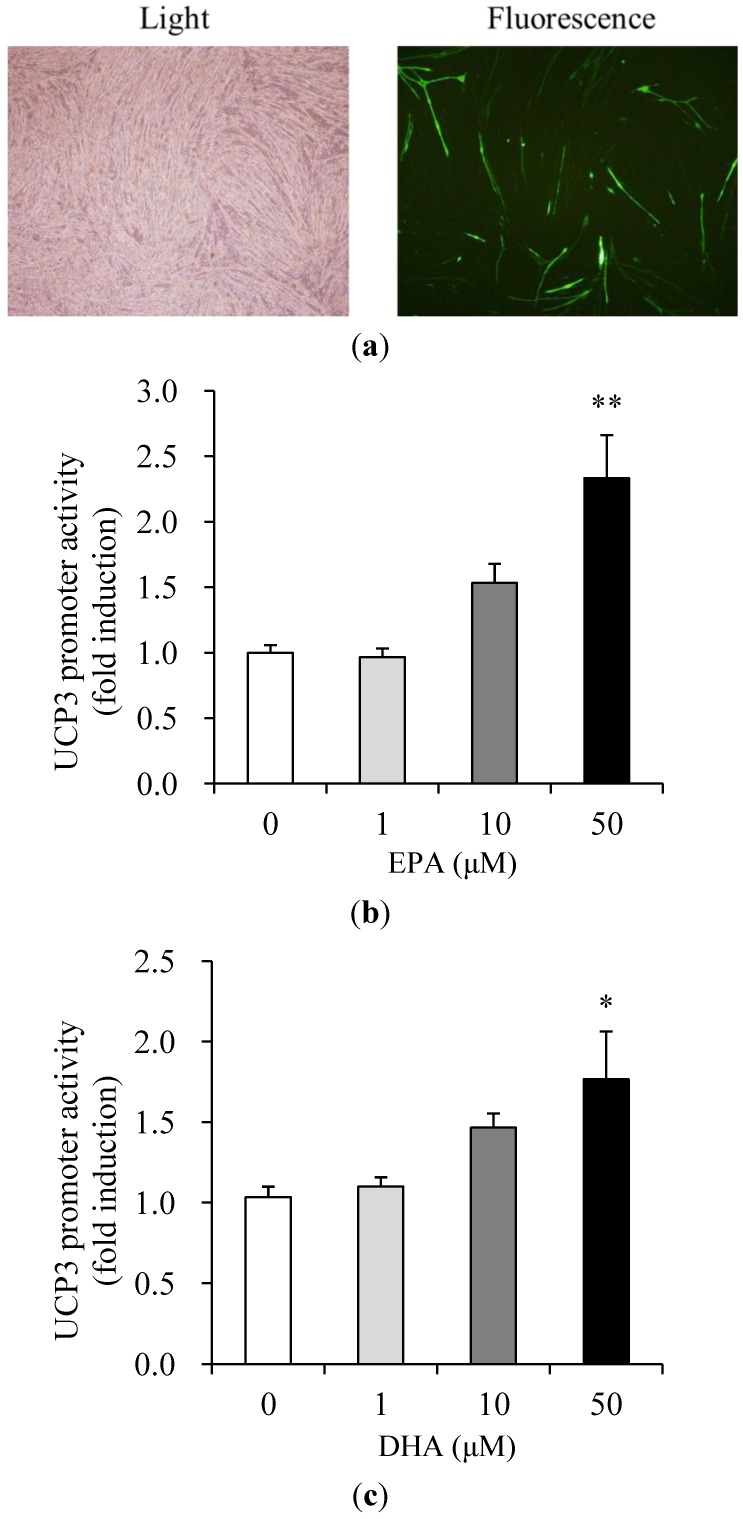
Effects of EPA and DHA on UCP3 promoter activity in muscle cells. Transfection efficiency (**a**) of differentiated C_2_C_12_ muscle cells was analyzed with a GFP expression vector. The GFP expression was observed under fluorescent microscope at 40× magnification. Cells were transfected with the UCP3 (−1790/+52 bp)/luc reporter gene and pCMV-β galactosidase, and were then incubated 1% BSA serum-free medium with the concentrations of EPA (**b**) and DHA (**c**) indicated, from 0 (control) to 50 μM, for 40 h. Promoter activities measured by luciferase activity were calculated in relative light units (RLU) and normalized to β-galactosidase activity. Values are expressed as mean ± SE (*n* = 3) of three independent experiments. * *P* < 0.05 and ** *P* < 0.01 *versus* control treatment.

To further verify the up-regulation of the UCP3 gene by EPA and DHA, we assayed the promoter activity in C_2_C_12_ muscle cells. In a transient transfection experiment, transfection efficiency was 38.4% as calculated by counting GFP-positive cells under the fluorescent microscope (Figure 3a). UCP3 promoter activity was increased by 1.6- and 2.3-fold in the presence of 10 and 50 μM of EPA, respectively, compared to untreated control (Figure 3b), and by 1.5- and 1.8-fold in the presence of 10 and 50 μM of DHA, respectively (Figure 3c). Cotransfection with the control vector (pGL3-basic) showed a negligible effect on luciferase activity (data not shown).

### 3.4. Effects of AICAR on EPA- and DHA—Stimulated UCP3 Promoter Activity

**Figure 4:**
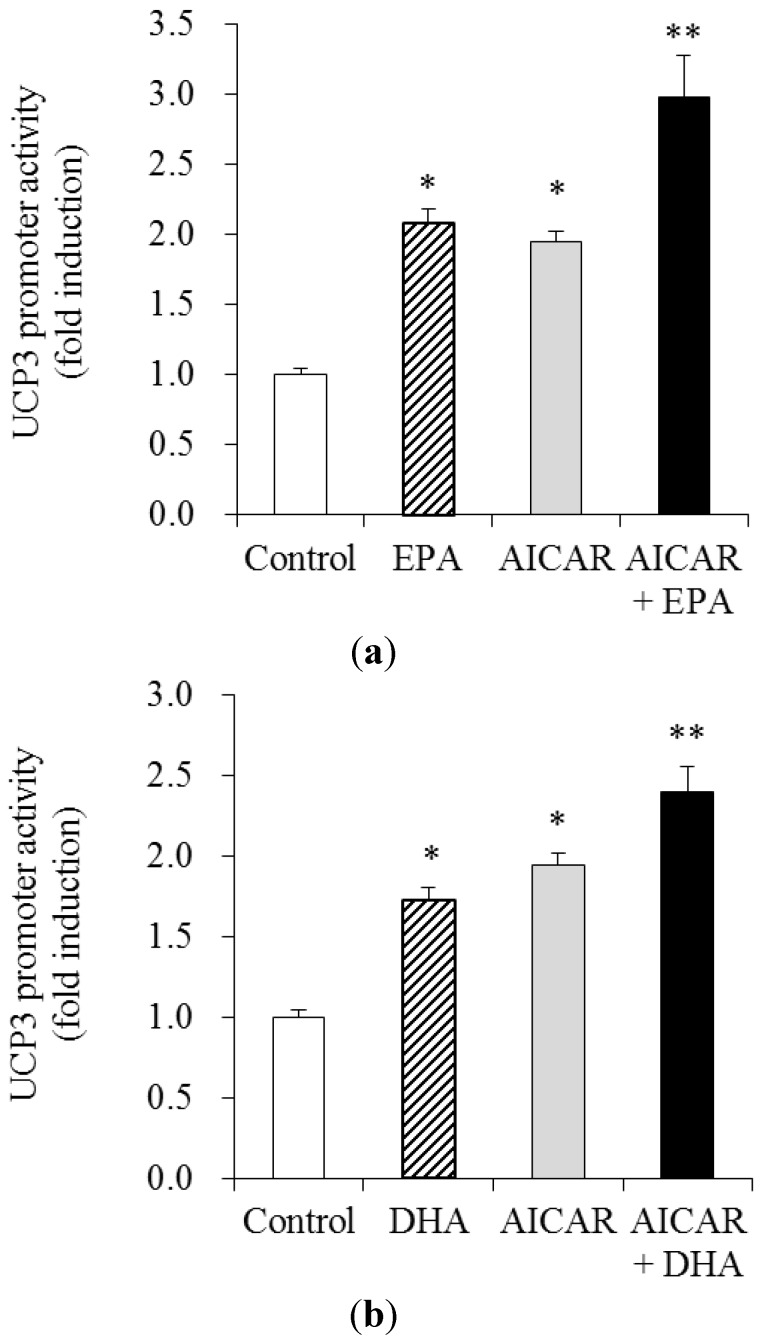
Effects of 5′-amino-4-imidazolecarboxamide-ribonucleoside (AICAR) on the EPA- and DHA-induced UCP3 promoter activity in muscle cells. Differentiated C_2_C_12_ muscle cells were transfected with the UCP3 (−1790/+52 bp)/luc reporter gene and pCMV-β galactosidase, were cultured in 1% BSA serum-free media for 40 h with 50 μM of EPA (**a**) and DHA (**b**) in the presence of 0.25 mM AICAR. Luciferase activity were calculated in relative light units (RLU) and normalized to β-galactosidase activity. Values are expressed as mean ± SE (*n* = 3) of three independent experiments. * *P* < 0.05 and ** *P* < 0.01 *versus* control treatment.

To investigate whether AMPK activation and PUFAs synergistically stimulate UCP3 expression, we determined the effects of EPA (50 μM) and DHA (50 μM) in combination with the AMPK stimulator AICAR (0.25 mM) on the UCP3 promoter activity in C_2_C_12_ muscle cells. Interestingly, cotreatment of EPA + AICAR further increased UCP3 promoter activity by 43% compared to EPA alone-treated cells (Figure 4a). Cotreatment of DHA Cotreatment of DHA +AICAR also further increased UCP3 promoter activity by 33% compared to DHA alone-treated cells (Figure 4b).

## 4. Discussion

The mitochondrial UCP3 exhibited an important role in regulating the energy balance of skeletal muscle under fasting and high-fat feeding conditions [15]. Consumption of a diet that contains omega-3 fatty acids (EPA and DHA) from fish oil and marine species confers a variety of benefits that include improved lipid metabolism and prevention of obesity and diabetes [16,17,18,19]. Moreover, fatty acids activate UCP3 expression when thermogenesis is required, which suggests that the activation of UCP3 by physiological activators might cause significant thermogenesis under certain conditions [20].

Here, we evaluated effect of EPA and DHA on the promoter activity and mRNA levels of UCP3 in C_2_C_12_ muscle cells. First, we investigated the potential cytotoxic effects of EPA and DHA on C_2_C_12_ muscle cells. Toxicity was unaffected in the concentrations of 1–50 μM of EPA and DHA. The plasma concentrations of EPA and DHA were between 0.2 and 0.42 mM, representing about 2%–4% of the total fatty acids (8.56–12.24 mM) [21]. Focusing specifically on the low concentrations of EPA and DHA, concentrations of 1–50 μM were used in our *in vitro* study.

Nuclear peroxisome proliferator-activated receptors (PPAR) are members of the nuclear hormone receptor superfamily that binds to specific DNA response-elements as heterodimers with the retinoid X receptor [22]. PPAR is activated by natural ligands, including fish oil enriched with polyunsaturated fatty acids (PUFAs), such as DHA and EPA [22]. Increased expression of the PPAR and PPAR activation by fatty acids has been reported to be associated with avian UCP3 mRNA up-regulation [23], with potential PPAR response elements in the avian UCP3 gene promoter [23,24]. The present study identified that EPA and DHA increased UCP3 expression in a dose-dependent manner in C_2_C_12_ muscle cells. These elevations might have been due to enhancement of transcription and/or an increase in mRNA stability. To further verify the up-regulation of the UCP3 mRNA by EPA and DHA, we assayed the promoter activity. UCP3 promoter activity was also significantly increased in a dose-dependent manner in EPA- and DHA-treated C_2_C_12_ muscle cells. Thus, it is highly probable that the effects of EPA and DHA on UCP3 gene expression occur at the level of transcription, although the response elements have yet to be identified in the −1790/+52 bp portion of the UCP3 gene promoter. 

In our study, cellular changes of UCP3 gene expression by EPA and DHA were evaluated. EPA and DHA are postulated to increase UCP3 mRNA through up-regulation of UCP3 transcription in C_2_C_12_ muscle cells. In rodents, dietary fish oil reduces body fat mass by increasing mitochondrial UCP3 and peroxisomal fatty acid oxidation in skeletal muscle [7]. Another study conducted on aged mice showed that long-term (four months) DHA supplementation decreased body weight and fat mass, in parallel with an increase in UCP3 mRNA level in skeletal muscle [8]. It is possible that the action of EPA and DHA in reducing body fat is related to an increase in energy expenditure that occurs upon the stimulation of UCP3 transcription *in vivo*. 

AMPK is an enzyme that regulates energy metabolism in the direction of catabolism [11]. AMPK activation can regulate energy metabolism that favors the inhibition of lipogenesis and increasing thermogenesis [11]. In parallel with activation of PPAR, EPA and DHA stimulates AMPK activity, an energy-sensing kinase that acts as a potential target for the treatment of obesity [12]. AMPK activation by AICAR increases mRNA expression of PPARa in cultured muscle cells and mouse skeletal muscle [25]. AMPK activation increases UCP3 mRNA and protein content by 1.6- and 3.3-fold in whole muscle extracts, respectively, after chronic AICAR injections *in vivo* [13]. These previous reports suggested the possible involvement of AMPK and PPAR on the up-regulation of UCP3 by EPA and DHA. Our present study demonstrated that EPA and DHA increased UCP3 mRNA. We used pharmacological reagents of AMPK, AICAR, to provide for the direct involvement of this kinase in UCP3 regulation in C_2_C_12_ muscle cells. The activation of AMPK, achieved by the addition of AICAR, increased UCP3 gene promoter activity. Moreover, AMPK activation with EPA or DHA showed the additive effect on the UCP3 promoter activity, suggesting a certain interaction of EPA or DHA with AMPK on the UCP3 gene expression.

## 5. Conclusions

The present study investigated the effects of EPA and DHA on UCP3 gene expression in C_2_C_12_ muscle cells. The results show that EPA and DHA increases UCP3 mRNA level in a dose-dependent manner and similarly increased UCP3 promoter activity in C_2_C_12_ muscle cells. Furthermore, AICAR showed synergistic effects with EPA or DHA on the UCP3 promoter activation. These findings suggest that EPA and DHA directly regulate the gene expression of UCP3, potentially through AMPK-mediated pathway in C_2_C_12_ muscle cells. Future investigation should be done on the elucidation of the regulation of UCP3 gene expression by other fatty acids in order to understand the specificity for EPA and DHA.
